# Incidence of cardiovascular events and mortality in Korean patients with chronic kidney disease

**DOI:** 10.1038/s41598-020-80877-y

**Published:** 2021-01-13

**Authors:** Hyunjin Ryu, Jayoun Kim, Eunjeong Kang, Yeji Hong, Dong-Wan Chae, Kyu Hun Choi, Seung Hyeok Han, Tae Hyun Yoo, Kyubeck Lee, Yong-Soo Kim, Wookyung Chung, Yun Kyu Oh, Soo Wan Kim, Yeong Hoon Kim, Su Ah Sung, Joongyub Lee, Sue K. Park, Curie Ahn, Kook-Hwan Oh, Representing KNOW-CKD Study Group

**Affiliations:** 1 Department of Internal Medicine, Seoul National University Hospital, Seoul, Republic of Korea; 2 Medical Research Collaborating Center, Seoul National University Hospital, Seoul, Republic of Korea; 3 Department of Internal Medicine, Ewha Womans University Medical Center, Seoul, Republic of Korea; 4 Rehabilitation Medical Research Center, Korea Workers’ Compensation and Welfare Service Incheon Hospital, Incheon, Republic of Korea; 5 Department of Internal Medicine, Seoul National University Bundang Hospital, Gyeonggi-do, Republic of Korea; 6 Department of Internal Medicine, Yonsei University College of Medicine, Seoul, Republic of Korea; 7 Department of Internal Medicine, Kangbuk Samsung Hospital, College of Medicine, Sungkyunkwan University, Seoul, Korea; 8 Department of Internal Medicine, The Catholic University of Korea College of Medicine, Seoul, Republic of Korea; 9 Department of Internal Medicine, Gachon University of Medicine and Science, Incheon, Republic of Korea; 10 Department of Internal Medicine, Seoul National University Boramae Medical Center, Seoul, Korea; 11 Department of Internal Medicine, Chonnam National University Medical School, Gwangju, Korea; 12 Department of Internal Medicine, Inje University College of Medicine, Busan, Korea; 13 Department of Internal Medicine, Eulji Medical Center, Eulji University, Seoul, Korea; 14 Department of Prevention and Management, Inha University Hospital, Incheon, Korea; 15 Department of Preventive Medicine, Seoul National University College of Medicine, Seoul, Korea; 16 Department of Internal Medicine, Seoul National University College of Medicine, Seoul, 03080 Republic of Korea

**Keywords:** Chronic kidney disease, Epidemiology

## Abstract

Few studies have investigated the incidence of cardiovascular disease (CVD) in the Asian chronic kidney disease (CKD) population. This study assessed the incidence of CVD, death, and a composite outcome of CVD and death in a prospective Korean predialysis CKD cohort. From a total of 2179 patients, incidence rates were analyzed, and competing risk analyses were conducted according to CKD stage. Additionally, incidence was compared to the general population. During a median 4.1 years of follow-up, the incidence of CVD, all-cause death, and the composite outcome was 17.2, 9.6, and 24.5 per 1000 person-years, respectively. These values were higher in diabetic vs. non-diabetic subjects (*P* < 0.001). For all outcomes, incidence rates increased with increasing CKD stage (CVD, *P* = 0.001; death, *P* < 0.001; and composite, *P* < 0.001). Additionally, CKD stage G4 [hazard ratio (HR) 2.8, *P* = 0.008] and G5 (HR 5.0, *P* < 0.001) were significant risk factors for the composite outcome compared to stage G1 after adjustment. Compared to the general population, the total cohort population (stages G1–G5) showed significantly higher risk of CVD (HR 2.4, *P* < 0.001) and the composite outcome (HR 1.7, *P* < 0.001). The results clearly demonstrate that CKD is a risk factor for CVD in an Asian population.

## Introduction

The prevalence and incidence of cardiovascular disease (CVD) and mortality increase as kidney function declines^1,2^. This knowledge was primarily based on population cohort studies, where patients with an estimated glomerular filtration rate (eGFR) < 60 mL/min/1.73 m^2^ had 2- to 16-fold higher risk of CVD compared to those with eGFR > 60 mL/min/1.73 m^2^^3,4^. Furthermore, the chronic kidney disease (CKD) population experiences increased risk of death, cardiovascular events, and hospitalization as baseline eGFR decreases^3,5^. Data from the Chronic Renal Insufficiency Cohort (CRIC) Study, which is a predialysis CKD prospective cohort in the US mainly composed of Caucasian (42%) and African American (42%) subjects, revealed an overall incidence of CVD and death of 38 per 1000 person-years (PYs) and 31 per 1000 PYs, respectively^6^.

Both traditional and non-traditional risk factors play a role in increased CVD in the CKD population^2^. The Kidney Disease Improving Global Outcome (KDIGO) guidelines for CKD recommended that all people with CKD be considered at increased risk for CVD and be assessed and treated for traditional risk factors, as in the general population^7^.

However, most previous studies on CVD incidence and risks in the CKD population were conducted in Western populations; the risk of atherosclerotic CVD is generally lower in the Asian-American population compared to non-Hispanic whites^8^. In addition, in the CKD population, several reports have shown that Asians have a lower incidence of CVD compared to Caucasians and African Americans, but these are mainly from US data^9,10^. A few studies have investigated the risk of cardiovascular events in the CKD population in Asian countries; however, most of these studies were based on subgroup analysis from the general population^11–14^. Recently, a report from the Chronic Kidney Disease Japan Cohort (CKD-JAC) study, which included a cohort of Japanese predialysis CKD patients with eGFR 10–59 mL/min/1.73 m^2^, showed the incidence of CVD to be 22.8 per 1000 PYs, which is lower than that reported from the CRIC study^15^. In the present study, we analyzed the incidence rates of CVD, all-cause death, and a composite outcome in a Korean predialysis CKD cohort, the KoreaN cohort Study for Outcome in Patients With CKD (KNOW-CKD). Additionally, the incidence of CVD and the composite outcome in the KNOW-CKD cohort were compared to those of the Korean general population.

## Materials and methods

### Study design and participants

**Figure 1:**
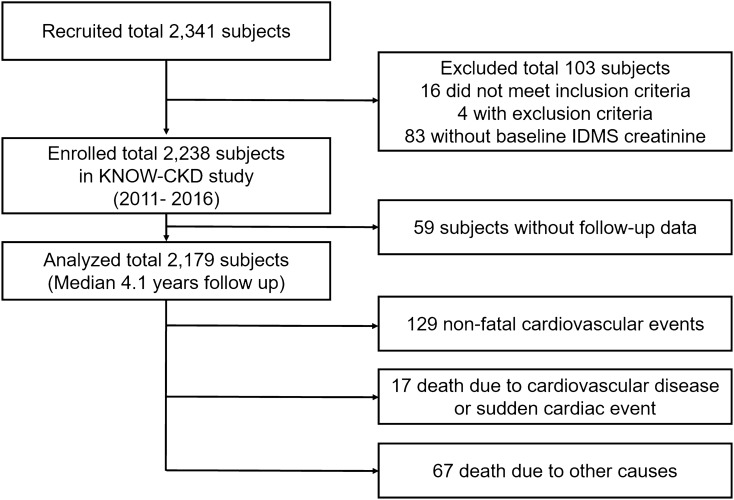
Flowchart of the enrolled study population.

This is a longitudinal study from a nationwide, prospective cohort of pre-dialysis CKD patients in Korea, entitled KNOW-CKD, which enrolled adult (20–75 years old) predialysis patients with CKD stages G1–G5 from nine centers between 2011 and 2016^16^. Of the 2238 subjects enrolled in the study, 59 patients without follow-up data were excluded; therefore, 2,179 patients were included in the final analysis (Fig. 1). Informed consent was obtained from all patients voluntarily at the time of enrollment. The study was approved by the institutional review board (IRB) of each participating hospital: Seoul National University Hospital (H-1704-025-842), Seoul National University Bundang Hospital (B-1106/129-008), Yonsei University Severance Hospital (4-2011-0163), Kangbuk Samsung Medical Center (2011-01-076), Seoul St. Mary’s Hospital (KC11OIMI0441), Gil Hospital (GIRBA2553), Eulji General Hospital (201105-01), Chonnam National University Hospital (CNUH-2011-092), and Pusan Paik Hospital (11-091). This study follows the guidelines of the 2008 Declaration of Helsinki.

### Data collection and measurements

Demographics and medical details including smoking history, comorbidities, cause of CKD, and medication history were collected at enrollment. Comorbidity of CVD was defined as any history of coronary artery disease, peripheral artery disease, cerebrovascular disease, or congestive heart failure. Patients previously diagnosed with diabetes or who were on diet modification or anti-diabetic medication were defined as diabetic patients. The following laboratory variables were measured using an 8-h fasting blood sample at each participating center laboratory: hemoglobin, fasting blood sugar, uric acid, calcium, phosphorous, albumin, total cholesterol, low-density lipoprotein cholesterol, high-density lipoprotein cholesterol, and high sensitivity C-reactive protein (hsCRP). Using a serum separation tube, 10 ml of whole blood was obtained and centrifuged within 1 h for serum separation, then sent to the central laboratory (Lab Genomics, Seoul, Republic of Korea) for measurement of creatinine and intact parathyroid hormone (iPTH). Serum creatinine was measured using an isotope dilution mass spectrometry-traceable method and serum iPTH was measured using chemiluminescence immunoassay. The CKD Epidemiology Collaboration equation based on serum creatinine was used to calculate eGFR^17^. For urine biochemistry, first-voided urine samples (15 ml) were collected and sent to the central laboratory for urine protein and creatinine assays. Urine protein and creatinine were measured using immunoturbidimetry and isotope dilution mass spectrometry-traceable methods, respectively. Patients were followed every year for renal and cardiovascular events. Death and causes of death were determined using either hospital medical records or the National Health Insurance System (NHIS) and Korea Statistical Information Service.

### Outcome definition

The primary outcome was CVD defined as any first event of the following: acute myocardial infarction, unstable angina, either ischemic or hemorrhagic cerebral stroke, congestive heart failure, symptomatic arrhythmia, aggravated valvular heart, pericardial disease, abdominal aortic aneurysm, and severe peripheral arterial disease that required hospitalization, intervention, or therapy during the follow-up. The secondary outcome was a composite of CVD and all-cause death. For the present study, data were collected until drop-out, death, or March 31, 2018, whichever came first.

### Statistical analysis

Baseline characteristics were compared according to the occurrence of outcomes using chi-square test and *t*-test methods. For non-normally distributed continuous variables, Mann–Whitney *U-*test was used. Incidence differences with respect to the diabetic condition and CKD stages were compared using Poisson regression analysis. To explore the effect of CKD stages on adverse outcomes, we developed cause-specific hazard models as the competing risk analysis with stage G1 as a reference^18^. Renal replacement therapy (RRT) prior to the outcome was considered a competing risk. Using variables that showed significant differences between CVD ( +) and CVD (−) groups and after removing variables that could act as mediators, adjusted models were constructed as follows: model 1, adjusted for age and sex; model 2, adjusted for the variables in model 1 plus diabetes and preexisting CVD; and model 3, further adjusted for diastolic blood pressure, total cholesterol, high-density lipoprotein cholesterol, and hsCRP. Sensitivity analysis was performed to determine the HR of outcomes in each CKD stage of G3a to G5 compared to combined stages G1–G2, to address the possibility that subjects with glomerular hyperfiltration might have been included in stage G1 in our CKD population. The results were presented as hazard ratios (HRs) and 95% confidence intervals (CIs). Subgroup analyses were performed according to age, sex, diabetes, urine protein-to-creatinine ratio of 1 g/g Cr, and cause of CKD. In subgroup analysis, HRs were derived from cause-specific hazard models adjusted for age and sex, with CKD stages G1–G2 as the reference. To compare the outcome incidence with the Korean general population, the NHIS—National Sample Cohort (2002–2013) was used ^19^. The sample cohort consists of a million subjects (2% of the total Korean population), and was selected as a representative sample using systematic stratified random sampling. After excluding patients with preexisting CVD events within 1 year, pregnancy, cancer, liver cirrhosis, organ transplantation, and RRT, a total of 710,362 subjects aged 20–79 were finally included in the comparative analysis (Supplemental Methods). Both unadjusted and age-and-sex adjusted Cox-proportional hazards models were conducted to calculate the HRs of CVD and the composite outcome for the KNOW-CKD cohort in comparison with the Korean general population. *P* < 0.05 was considered statistically significant in all statistical analyses. All statistical analyses were conducted using SAS 9.4 (SAS Institute, Cary, NC) and R version 3.6.1 (Foundation for Statistical Computing, Vienna, Austria).

## Results

A total of 2179 patients were included in this study (Fig. 1), 38.7% of whom were female, with a mean age of 53.6 ± 12.2 years, and a mean eGFR of 53.2 ± 30.7 mL/min/1.73 m^2^. In total, 16.2% were CKD stage G1, 19% stage G2, 16.5% stage G3a, 21.1% stage G3b, 21.2% stage G4, and 6.1% stage G5. Among the total study population, 6.0% had a preexisting history of CVD, and 33.4% were diabetic.

During the median 4.1 [interquartile range, 2.8–5.8] years of follow-up, a total of 146 cases of CVD occurred, among which 129 were non-fatal. In addition, 84 cases of death occurred during follow-up due to the following causes: CVD (n = 15), infection (n = 17), malignancy (n = 10), sudden cardiac death (n = 2), liver disease (n = 3), other causes (n = 11), and cause unknown (n = 26). The composite of CVD and death occurred in 208 patients in the study population.

The incidence rates of CVD, all-cause death, and the composite outcome were 17.2, 9.6, and 24.5 per 1000 PYs, respectively. The incidence rates of specific types of CVD are detailed in Table 1 and Supplementary Figure S1. The incidence of CVD (29.7 vs. 11.6 per 1000 PYs, *P* < 0.001), death (16.6 vs. 6.4 per 1000 PYs, *P* < 0.001), and the composite outcome (41.5 vs. 17.0 per 1000 PYs, *P* < 0.001) was higher in diabetics than in non-diabetics. When we compared CKD stages G1–G2 to stages G3a–G5, the incidence of CVD (11.0 vs. 20.6 per 1000 PYs, *P* < 0.001), death (2.8 vs. 13.3 per 1000 PYs, *P* < 0.001), and the composite outcome (13.5 vs. 30.7 per 1000 PYs, *P* < 0.001) was higher in the stage G3a–G5 group (Table 1).

**Table 1:** Incidence of cardiovascular disease, death, and composite outcome in the KNOW-CKD cohort.

	Total (n = 2179)		Diabetes (n = 728)		Non-diabetes (n = 1451)		P-value	CKD stage G1-G2 (n = 767)		CKD stage G3a-G5 (n = 1412)		P-value
	Event number	Incidence^a^	Event number	Incidence^a^	Event number	Incidence^a^		Event number	Incidence^a^	Event number	Incidence^a^	
Composite outcome	208	24.5	107	41.5	101	17.0	< 0.001	42	13.5	166	30.7	< 0.001
Death	84	9.6	45	16.6	39	6.4	< 0.001	9	2.8	75	13.3	< 0.001
Cardiovascular disease	146	17.2	77	29.7	69	11.6	< 0.001	34	11.0	112	20.6	0.001
Coronary artery disease	72	8.4	44	17.0	28	4.7	< 0.001	12	3.9	60	11.0	0.001
Ischemic stroke	24	2.8	15	5.8	9	1.5	0.001	6	1.9	18	3.3	0.254
Congestive heart failure	5	0.6	4	1.5	1	0.2	0.047	1	0.3	4	0.7	0.460
Cerebral hemorrhage	9	1.1	4	1.5	5	0.8	0.366	5	1.6	4	0.7	0.242
Others	36	4.2	10	3.9	26	4.4	0.736	10	3.2	26	4.8	0.289
Follow up time (person-year)	8794.4		2718.1		6076.3			3176.2		5618.1		

P-value from Poisson regression model in the incidence comparisons of diabetes vs. non-diabetes and CKD stages G1–G2 vs. CKD stages G3a–G5.

*CKD* chronic kidney disease.

^a^Incidence per 1000 person-years.

Table 2 and Supplementary Table S1 compare the baseline clinical characteristics between subgroups who developed and did not develop any CVD or composite events. The CVD ( +) group was older (61.2 ± 8.4 years vs. 53.1 ± 12.3 years, *P* < 0.001) and included more male patients (72.9% vs. 60.6%, *P* = 0.006) compared to the CVD (−) group. In addition, in the CVD ( +) group, diastolic blood pressure was lower (74.9 ± 11.6 vs. 77.0 ± 11.0, *P* = 0.028), left ventricular mass index was higher (105.4 ± 30.6 vs. 92.9 ± 24.5, *P* < 0.001), and the prevalence of preexisting CVD was higher (24% vs. 5.2%, *P* < 0.001). The CVD ( +) group included more diabetic subjects (52.7% vs. 32%, *P* < 0.001), and causes of CKD differed between the CVD ( +) and the CVD (−) groups (*P* < 0.001). eGFR for the CVD ( +) group was 43.9 ± 22.2 mL/min/1.73 m^2^, which was lower than that of the CVD (−) group (53.9 ± 31.2 mL/min/1.73 m^2^) (*P* < 0.001). In addition to factors associated with CVD occurrence, various other laboratory measures showed significant differences according to composite outcome occurrence (Supplementary Table S1).

**Table 2:** Comparison of baseline clinical characteristics at enrollment with respect to the occurrence of cardiovascular disease.

	Total	CVD ( +)	CVD (−)	P-value
Number	2179	146 (6.7)	2033 (93.3)	
Age (years)	53.6 ± 12.2	61.2 ± 8.4	53.1 ± 12.3	< 0.001
Female, n (%)	843 (38.7)	41 (28.1)	802 (39.4)	0.006
BMI, (kg/m^2^)	24.6 ± 3.4	24.6 ± 2.9	24.6 ± 3.4	0.81
Systolic blood pressure (mmHg)	128.5 ± 16.5	129.9 ± 17.4	128.4 ± 16.4	0.28
Diastolic blood pressure (mmHg)	76.9 ± 11.1	74.9 ± 11.6	77.0 ± 11.0	0.03
Waist to hip ratio	40.5 ± 6.1	40.4 ± 4.9	40.5 ± 6.2	0.78
Left ventricular mass index (g/m^2^)	93.8 ± 25.1	105.4 ± 30.6	92.9 ± 24.5	< 0.001
Prevalence of cardiovascular disease, n (%)	140 (6.4)	35 (24)	105 (5.2)	< 0.001
Prevalence of diabetic mellitus, n (%)	728 (33.4)	77 (52.7)	651 (32)	< 0.001
**Cause of chronic kidney disease**				< 0.001
Glomerulonephritis, n (%)	789 (36.2)	29 (19.9)	760 (37.4)	
Diabetic mellitus, n (%)	502 (23)	61 (41.8)	441 (21.7)	
Hypertension, n (%)	401 (18.4)	27 (18.5)	374 (18.4)	
Polycystic kidney disease, n (%)	357 (16.4)	14 (9.6)	343 (16.9)	
Others, n (%)	130 (6)	15 (10.3)	115 (5.7)	
eGFR (mL/min/1.73 m^2^)	53.2 ± 30.7	43.9 ± 22.2	53.9 ± 31.2	< 0.001
**Chronic kidney disease stage**				0.002
G1, n (%)	352 (16.2)	7 (4.8)	345 (17.0)	
G2, n (%)	415 (19)	27 (18.5)	388 (19.1)	
G3a, n (%)	359 (16.5)	24 (16.4)	335 (16.5)	
G3b, n (%)	459 (21.1)	42 (28.8)	417 (20.5)	
G4, n (%)	462 (21.2)	38 (26.0)	424 (20.9)	
G5, n (%)	132 (6.1)	8 (5.5)	124 (6.1)	
Hemoglobin (g/dL)	12.8 ± 2.0	12.5 ± 2.1	12.9 ± 2.0	0.03
Uric acid (mg/dL)	7.0 ± 1.9	7.2 ± 1.9	7.0 ± 1.9	0.35
Calcium (mg/dL)	9.1 ± 0.5	9.0 ± 0.5	9.1 ± 0.5	0.06
Phosphorous (mg/dL)	3.7 ± 0.7	3.7 ± 0.8	3.7 ± 0.7	0.57
Albumin (g/dL)	4.2 ± 0.4	4.2 ± 0.4	4.2 ± 0.4	0.11
Total cholesterol (mg/dL)	174.0 ± 39.2	167.1 ± 36.9	174.5 ± 39.3	0.03
HDL-cholesterol (mg/dL)	49.2 ± 15.3	46.0 ± 12.9	49.5 ± 15.5	0.002
LDL-cholesterol (mg/dL)	96.9 ± 31.8	92.3 ± 28.0	97.2 ± 32.0	0.08
Triglyceride (mg/dL)	157.3 ± 98.6	160.2 ± 89.1	157.1 ± 99.2	0.72
Fasting blood sugar (mg/dL)	111.0 ± 39.8	119.2 ± 50.6	110.4 ± 38.8	0.01
HbA1C (%) (only in diabetic patients)	7.2 ± 1.3	7.2 ± 1.1	7.2 ± 1.3	0.99
Parathyroid hormone (pg/mL)	51.1 (32.3–84.0)	56.0 (37.0–100.0)	51.0 (32.0–83.2)	0.06
Urine protein/creatinine (g/g Cr)	0.5 (0.1–1.5)	0.7 (0.2–2.2)	0.5 (0.1–1.5)	0.01
Hs-CRP (mg/dL)	0.6 (0.2–1.7)	0.8 (0.3–2.0)	0.6 (0.2–1.6)	0.02
Follow up duration (years)	4.0 ± 1.7	4.1 ± 1.8	4.0 ± 1.7	0.55

*BMI* body mass index, *CVD* cardiovascular disease, *eGFR* estimated glomerular filtration rate, *HbA1C* hemoglobin A1C, *HDL* high-density lipid, *Hs-CRP* high sensitivity-C reactive protein, *LDL* low-density lipid.

**Figure 2:**
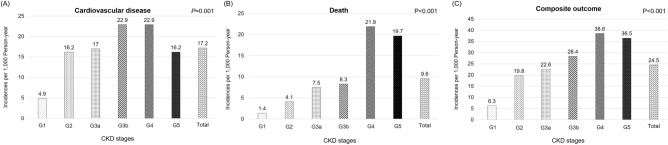
Incidence of outcomes according to chronic kidney disease stage in the KNOW-CKD cohort. *CKD* chronic kidney disease.

The incidence of CVD, all-cause death, and composite outcome increased with increasing CKD stage from G1 to G4 (*P* = 0.001, *P* < 0.001, and *P* < 0.001, respectively, Fig. 2). However, in predialysis stage G5, the incidence of CVD, death, and composite outcome all dropped compared to stage G4. The incidence of specific CV types according to CKD stage are shown in Supplementary Fig. 1.

To investigate the effects of baseline CKD stage on outcome occurrence in the cohort population, competing risk analysis was employed. With the unadjusted model, a significantly increased HR for CVD was observed in stages G2, G3a, G3b, G4, and G5 (HRs of 3.44 [95% CI, 3.76, 5.26, 6.23, and 7.12, respectively]) compared to stage G1. However, in model 3, the statistical significance disappeared. The unadjusted model showed an increased HR for the composite outcome in stages G2, G3a, G3b, G4, and G5 (HRs of 3.34, 4.01, 5.19, 8.76, and 13.19, respectively). In fully adjusted model 3, CKD stages G4 and G5 showed significantly increased HRs of 2.79 (95% CI 1.31–5.95, *P* = 0.008) and 4.95 (95% CI 2.10–11.67, *P* < 0.001), respectively (Table 3).

**Table 3:** Cause-specific hazard model for the multivariate regression analysis of outcomes according to CKD stages after adjustment with confounding factors (CKD stage G1 as reference).

CKD stages	Unadjusted		Model 1		Model 2		Model 3	
	HR (95% CI)	P-value	HR (95% CI)	P-value	HR (95% CI)	P-value	HR (95% CI)	P-value
**Primary outcome: cardiovascular disease**								
Stage G1	Reference		Reference		Reference		Reference	
Stage G2	3.44 (1.50–7.91)	0.004	2.05 (0.88–4.77)	0.094	2.09 (0.90–4.86)	0.088	1.91 (0.81–4.51)	0.139
Stage G3a	3.76 (1.62–8.74)	0.002	1.68 (0.70–4.00)	0.244	1.54 (0.64–3.69)	0.333	1.39 (0.57–3.39)	0.472
Stage G3b	5.26 (2.36–11.74)	< 0.001	2.26 (0.98–5.19)	0.055	1.88 (0.81–4.38)	0.142	1.81 (0.77–4.27)	0.177
Stage G4	6.23 (2.77–14.02)	< 0.001	2.49 (1.07–5.79)	0.034	2.02 (0.86–4.74)	0.107	1.87 (0.78–4.52)	0.163
Stage G5	7.12 (2.57–19.78)	< 0.001	3.21 (1.12–9.19)	0.029	2.99 (1.05–8.50)	0.041	2.66 (0.89–8.00)	0.081
**Secondary outcome: composite outcome of cardiovascular disease and all-cause death**								
Stage G1	Reference		Reference		Reference		Reference	
Stage G2	3.34 (1.60–6.99)	0.001	2.01 (0.95–4.24)	0.067	2.04 (0.97–4.31)	0.062	1.83 (0.86–3.92)	0.118
Stage G3a	4.01 (1.91–8.42)	< 0.001	1.79 (0.84–3.84)	0.133	1.65 (0.77–3.55)	0.198	1.49 (0.68–3.25)	0.316
Stage G3b	5.19 (2.55–10.55)	< 0.001	2.25 (1.08–4.69)	0.030	1.93 (0.92–4.05)	0.083	1.90 (0.90–4.04)	0.095
Stage G4	8.76 (4.34–17.66)	< 0.001	3.46 (1.67–7.20)	< 0.001	2.90 (1.38–6.06)	0.005	2.79 (1.31–5.95)	0.008
Stage G5	13.19 (5.89–29.52)	< 0.001	5.66 (2.46–13.03)	< 0.001	5.24 (2.28–12.06)	< 0.001	4.95 (2.10–11.67)	< 0.001

Model 1: adjusted for age and sex.

Model 2: adjusted for variables in model 1, in addition to prevalence of diabetes, and prevalence of cardiovascular disease.

Model 3: adjusted for variables in model 2, in addition to diastolic blood pressure, total cholesterol, HDL cholesterol, and high-sensitivity C-reactive protein.

*CI* confidential interval, *CKD* chronic kidney disease, *HR* hazard ratio.

Sensitivity analysis was conducted to determine the HR of outcomes in stages G3a, G3b, G4, and G5 compared to stages G1–G2 together as a reference (Supplementary Table S2). Results were similar to the initial findings.

Subgroup analysis was conducted according to age, sex, prevalence of diabetes, proteinuria, and CKD causes. In the age- and sex-adjusted cause-specific hazard model, the HRs for composite outcome were significantly increased at stages G4–G5 compared to stages G1–G2 in the following subgroups: aged ≥ 55 years, males, both with and without diabetes, urine protein-to-creatinine ratio < 1 g/g Cr, and polycystic kidney disease (Supplementary Table S3).

To elucidate the risk of CVD or the composite outcome of predialysis CKD subjects compared to the Korean general population, multivariate Cox regression analysis was conducted to determine the HR of CVD and the composite outcome in the KNOW-CKD population compared to the general population (NHIS-National Sample Cohort) after adjusting for age and sex. The risk of CVD and the composite outcome was higher in the KNOW-CKD cohort compared to the general population; HRs were 2.35 (95% CI 1.99–2.76, *P* < 0.001) and 1.67 (95% CI 1.46–1.91, *P* < 0.001), respectively. All CKD stages except for G1 and G5 showed an approximately two-fold increased risk of CVD. Meanwhile, for the composite outcome, only stages G2, G3b, G4, and G5 showed significantly increased HRs (Table 4).

**Table 4:** Age and sex adjusted hazard ratios of outcome risks in the KNOW-CKD cohort compared to the general population using multivariate Cox regression analysis.

CKD stages	Cardiovascular disease				Composite outcome			
	Unadjusted		Age, sex adjusted		Unadjusted		Age, sex adjusted	
	HR (95% CI)	P-value	HR (95% CI)	P-value	HR (95% CI)	P-value	HR (95% CI)	P-value
General population	Reference		Reference		Reference		Reference	
All stages	4.60 (3.91, 5.42)	< 0.001	2.35 (1.99, 2.76)	< 0.001	3.54 (3.09, 4.05)	< 0.001	1.67 (1.46, 1.91)	< 0.001
Stage G1	1.39 (0.68, 2.85)	0.363	1.77 (0.87, 3.62)	0.118	0.95 (0.50, 1.79)	0.866	1.19 (0.63, 2.26)	0.584
Stage G2	4.41 (3.03, 6.41)	< 0.001	2.87 (1.98, 4.18)	< 0.001	2.88 (2.05, 4.05)	< 0.001	1.77 (1.26, 2.48)	0.001
Stage G3a	4.63 (3.11, 6.88)	< 0.001	2.01 (1.35, 2.99)	< 0.001	3.31 (2.35, 4.67)	< 0.001	1.33 (0.95, 1.87)	0.103
Stage G3b	6.23 (4.62, 8.41)	< 0.001	2.62 (1.94, 3.54)	< 0.001	4.10 (3.13, 5.37)	< 0.001	1.61 (1.23, 2.11)	< 0.001
Stage G4	6.26 (4.57, 8.58)	< 0.001	2.44 (1.78, 3.35)	< 0.001	5.60 (4.38, 7.15)	< 0.001	2.03 (1.59, 2.59)	< 0.001
Stage G5	4.66 (2.39, 9.09)	< 0.001	1.91 (0.98, 3.75)	0.059	5.37 (3.41, 8.48)	< 0.001	2.12 (1.35, 3.34)	0.001

*CI* confidential interval, *CKD* chronic kidney disease, *HR* hazard ratio, *UPCR* urine protein-to-creatinine ratio.

## Discussion

The KNOW-CKD cohort was designed to discover outcomes and complications in the Korean predialysis CKD population and to investigate their associated risk factors. Here, we report incidence rates of CVD, death, and the composite outcome of 24.5, 9.6, and 17.2, respectively, per 1,000 PYs among overall CKD subjects. Previous studies conducted in a Western CKD population reported that patients with CKD stage G3–G5 have a higher risk of death compared to the risk of progression to end-stage renal disease^20^. However, in our population, the incidence of CVD, death, and the composite outcome was much lower compared to end-stage renal disease development, which was 57.6 per 1000 PYs. This is similar to results seen among Japanese CKD subjects^15^.

Supplementary Figure S2 shows a comparison of the incidence of major outcomes among Korean and other major CKD cohorts, such as CKD-JAC, CRIC, and the African American Study of Kidney Disease and Hypertension (AASK). For comparison with other cohorts, we further selected only CKD stage G3a-G5 for analysis. When compared with the Japanese CKD cohort, the Korean subpopulation in the present study with stage G3a–G5 showed a similar incidence of CVD but a higher incidence of all-cause death. Although the overall incidence of CVD was similar, the types differed from those of the CKD-JAC population. However, the overall incidence of CVD (20.6 events per 1000 PYs) and death (13.3 events per 1000 PYs) was lower in the Korean cohort than in the CRIC and AASK populations.

We additionally showed that the incidence of CVD and the composite outcome in the Korean CKD cohort was higher than in the general population. Additionally, incidence rates of CVD, death, and the composite outcome were higher in diabetic and CKD stage G3–G5 patients. This indicates that CKD is a significant risk factor for CVD in the Korean population.

The different incidences of CVD between Asian and Western predialysis CKD patients might be attributed to different genetic backgrounds, diet, and lifestyles between Asian and Western countries^21,22^. Furthermore, the lower incidence and better control of traditional risk factors (obesity, hypertension, and diabetes) among Koreans might have resulted in the lower incidence of CVD^23–25^. However, the different baseline profiles might also have contributed. For example, the KNOW-CKD population also includes polycystic kidney disease patients, whereas they were excluded from other cohorts such as CKD-JAC and CRIC. To determine the meaningful differences in cardiovascular outcomes between different CKD cohorts, international comparative research is warranted. This would help elucidate both common and unique risk factors associated with major outcomes of CKD^25^.

Our study has several strengths. We included subjects at CKD stages G1 and G2 in the cohort population. Therefore, thorough risk stratification analysis was possible according to CKD stages within the cohort population itself. In this study, we demonstrated that the incidence of the composite outcome was significantly increased in stages G4 and G5 compared to stage G1 (Table 3) or stage G1–G2 (Supplementary Table S2). This result indicates that advanced CKD stage is an important risk factor for the composite outcome. In particular, the significance of advanced CKD stage was highlighted for risk factors such as age ≥ 55 years and male sex (Supplementary Table S3). The significance of advanced CKD stages is evident in both diabetic and non-diabetic patients even though patients with diabetes showed a higher incidence of outcomes. Although CKD stages G4–G5 only showed significantly increased HRs in patients with a urine protein-to-creatinine ratio < 1 g/g Cr, trends in increasing HR for stages G4–G5 were also seen in subgroups with a urine protein-to-creatinine ratio ≥ 1 g/g Cr (HR 1.76 [95% CI 0.94–3.29, *P* = 0.079]). Finally, by using the Korean general population data, we determined that overall risks of CVD and the composite outcome were significantly increased in the Korean predialysis CKD population.

A few limitations exist. First, only small numbers of CVD (6.7%) and composite events (9.5%) occurred in the KNOW-CKD cohort, which might have attenuated the statistical power of the analysis. However, the incidence of CVD was similar to that of Japan, which would support the relatively low risk of CVD in the Asian CKD population compared to Caucasian or African American ethnicities. Second, there might have been undetected CVD after starting RRT. However, we tried to account for this issue by performing competing risk analysis with RRT as the competing risk. Third, selection bias might have affected the results, since the KNOW-CKD cohort excluded unstable CKD stage G5 subjects who were expected to start RRT within six months and subjects with severe heart failure symptoms (New York Heart Association class 3–4). This could explain the relatively lower incidence of outcomes in stage G5 compared to stage G4. Fourth, since this is a multinational observational study that enrolled patients for five years, not all variable measurement methods can be unified. Critical variables such as serum creatinine, iPTH, and urine protein and creatinine were measured at a central laboratory. However other variables such as hemoglobin, uric acid, calcium, phosphorous, albumin, lipid panels, and high sensitivity C reactive protein were measured at each center. Also, inter-observer bias might exist since echocardiography was conducted at each center. Sixth, outcome definition and study period differed between the KNOW-CKD cohort and the Korean general population cohort (NHIS-National Sample Cohort). Therefore, more well-designed systematic studies are needed to seek out the standardized incidence ratio for CVD in the CKD population. However, the results of the present study are robust in that the incidence of CVD and the composite outcome was analyzed in a well-designed CKD cohort population that was systematically and prospectively followed. We have also shown that, among the included CKD stages G1 and G2 subgroup, composite outcome risk was significantly increased in stages G4 and G5 in the predialysis CKD population. When the primary outcome was compared with the Korean general population, the risk of CVD and the composite outcome increased significantly, even from CKD stage G2. Therefore, this study provides useful information on CVD incidence in the Asian CKD population in comparison with other ethnicities, and can act as the foundation for future studies investigating cardiovascular complications to determine risk factors unique to the Asian CKD population.

Using the KNOW-CKD cohort, we demonstrated that the incidence of CVD, death, and the composite outcome was 17.2, 9.6, and 24.5 per 1000 PYs, respectively. The incidence of the composite outcome increased in stages G4 and G5 compared to stage G1. Our Korean CKD cohort showed significantly increased risk of CVD and the composite outcome compared to the Korean general population. Therefore, managing both traditional and non-traditional risk factors of CVD is important in the Korean predialysis CKD population, especially in high-risk patients such as older patients and those with diabetes or advanced-stage CKD.

## Supplementary Information

Supplementary Information.
