# Author Correction: Incidence of cardiovascular events and mortality in Korean patients with chronic kidney disease

**DOI:** 10.1038/s41598-021-88996-w

**Published:** 2021-04-28

**Authors:** Hyunjin Ryu, Jayoun Kim, Eunjeong Kang, Yeji Hong, Dong‑Wan Chae, Kyu Hun Choi, Seung Hyeok Han, Tae Hyun Yoo, Kyubeck Lee, Yong‑Soo Kim, Wookyung Chung, Yun Kyu Oh, Soo Wan Kim, Yeong Hoon Kim, Su Ah Sung, Joongyub Lee, Sue K. Park, Curie Ahn, Kook‑Hwan Oh, Representing KNOW‑CKD Study Group

**Affiliations:** 1 Department of Internal Medicine, Seoul National University Hospital, Seoul, Republic of Korea; 2 Medical Research Collaborating Center, Seoul National University Hospital, Seoul, Republic of Korea; 3 Department of Internal Medicine, Ewha Womans University Medical Center, Seoul, Republic of Korea; 4 Rehabilitation Medical Research Center, Korea Workers’ Compensation and Welfare Service Incheon Hospital, Incheon, Republic of Korea; 5 Department of Internal Medicine, Seoul National University Bundang Hospital, Gyeonggi-do, Republic of Korea; 6 Department of Internal Medicine, Yonsei University College of Medicine, Seoul, Republic of Korea; 7 Department of Internal Medicine, Kangbuk Samsung Hospital, College of Medicine, Sungkyunkwan University, Seoul, Korea; 8 Department of Internal Medicine, The Catholic University of Korea College of Medicine, Seoul, Republic of Korea; 9 Department of Internal Medicine, Gachon University of Medicine and Science, Incheon, Republic of Korea; 10 Department of Internal Medicine, Seoul National University Boramae Medical Center, Seoul, Korea; 11 Department of Internal Medicine, Chonnam National University Medical School, Gwangju, Korea; 12 Department of Internal Medicine, Inje University College of Medicine, Busan, Korea; 13 Department of Internal Medicine, Eulji Medical Center, Eulji University, Seoul, Korea; 14 Department of Prevention and Management, Inha University Hospital, Incheon, Korea; 15 Department of Preventive Medicine, Seoul National University College of Medicine, Seoul, Korea; 16 Department of Internal Medicine, Seoul National University College of Medicine, Seoul, 03080 Republic of Korea

Correction to: *Scientific Reports* 10.1038/s41598-020-80877-y, published online 13 January 2021

This Article contains errors.

Firstly, the Acknowledgements section in this Article was omitted. The Acknowledgements section should read:

“The authors also thank the KNOW-CKD investigators, clinical research nurses, and the patients participating in the study.”

Additionally, the Data Availability section in this Article was omitted. The Data Availability section should read:

“All produced data are available as upon request.”

Finally, the Funding section in this Article was omitted. The Funding section should read:

“This work was supported by the Research Program funded by the Korea Centers for Disease Control and Prevention (2011E3300300, 2012E3301100, 2013E3301600, 2013E3301601, 2013E3301602, 2016E3300200, 2016E3300201, 2016E3300202, and 2019E320100).”

