# Supplementary material for: Incidence of cardiovascular events and mortality in Korean patients with chronic kidney disease
Source: Sci Rep. 2021 Jan 13;11:1131. doi: 10.1038/s41598-020-80877-y (PMC7806882; doi:10.1038/s41598-020-80877-y)
Supplement: Supplementary file 1 — Supplementary Information. [file 41598_2020_80877_MOESM1_ESM.docx]

**Incidence of Cardiovascular Events and Mortality in Korean Patients with Chronic Kidney Disease**

Hyunjin Ryu^1^ , Jayoun Kim^2^, Eunjeong Kang^3^, Yeji Hong^4^, Dong-Wan Chae^5^, Kyu Hun Choi^6^, Seung Hyeok Han^6^, Tae Hyun Yoo^6^, Kyubeck Lee^7^, Yong-Soo Kim^8^, Wookyung Chung^9^, Yun Kyu Oh^10^, Soo Wan Kim^11^, Yeong Hoon Kim^12^, Su Ah Sung^13^, Joongyub Lee^14^, Sue K. Park^15^, Curie Ahn^1, 16^, and Kook-Hwan Oh^1, 16^† Representing KNOW-CKD Study Group

**Supplementary Material**

**Supplementary Methods.** Methods for the comparison analysis between CKD cohort population and general population

**Supplementary Figure S1. Incidence of cardiovascular disease in the KNOW-CKD cohort according to CKD stage**

**Supplementary Figure S2. Incidence of outcomes compared to the other nations’ CKD cohorts**

**Supplementary Table S1. Comparison of baseline clinical characteristics at enrollment with respect to the occurrence of composite outcomes**

**Supplementary Table S2. Cause-specific hazard model for the multivariate regression analysis of outcomes according to CKD stage after adjustment for confounding factors (CKD stage 1 and 2 as a reference)**

**Supplementary Table S3. Age- and sex-adjusted hazard ratios according to CKD stage in subgroup analysis using a cause-specific hazard model (CKD stages G1-G2 as a reference)**

**Supplementary Methods.** Methods for the comparison analysis between CKD cohort population and general population

For the comparison analysis between CKD cohort population and general population in MACE and composite outcome, the National Health Insurance Service (NHIS) – National Sample Cohort (2002~2013) of 1 million population was used**.**

1. **Study subjects**

Among adult subjects of age between 20–79 years old (n=734,449), subjects with following criteria were excluded

1. Subjects with inpatient’s diagnosis (any of main diagnosis or sub diagnosis) of myocardial infarction (I21), unstable angina (I20.0), stroke (I60-I63), or congestive heart failure (I50) in year 2002.
2. Subjects with inpatient’s or outpatient’s diagnosis (any of main diagnosis or sub diagnosis) of pregnancy (O00-O08, O10-O16, O60-O75, O80-84, O85-O92, O94-O99), cancer (C00-C97), liver cirrhosis (K74) or organ transplantation (Z94) between year 2002 and 2013**.**
3. Subjects with inpatient’s or outpatient’s diagnosis (any of main diagnosis or sub diagnosis) of dialysis (Z49), or reimbursement code of hemodialysis or peritoneal diagnosis (O7020, O7021, O7073) between year 2002 and 2013**.**

After exclusion of total 24,087 subjects, total 710,362 subjects were analyzed for the study.

1. **Outcome definitions**

CVD was defined as the first event of hospitalization with inpatient’s diagnosis (main diagnosis or 1st sub diagnosis) of ICD-10 code I00—I99 during the follow-up (Virani SS et al., *Circulation*. 2020;141(9):e139-e596., and Kim MY et al., *J Korean Med Sci*. 2013 Sep;28(9):1316-22.). Death and causes of death were provided from Korea Statistical Information Service. Starting from January 1^st^ 2003, the study subjects were followed up until death, or December 31, 2013, whichever came first.

1. **Statistics**

Cox proportional hazard ratio was used for the statistical analysis to calculate the hazard ratio of the outcome in cohort CKD population compared to general population data. Both unadjusted and age-and-sex adjusted Cox regression model was conducted for the analysis.

1. **Baseline clinical characteristics of the study population among the general population cohort.**

|  | | **Total** |
| --- | --- | --- |
| **Number (%)** | | 710,362 |
| **Female, n (%)** | | 354,252 (49.9) |
| **Age (years)** | |  |
|  | 20-29, n (%) | 159,051 (22.4) |
|  | 30-39, n (%) | 179,084 (25.2) |
|  | 40-49, n (%) | 167,628 (23.6) |
|  | 50-59, n (%) | 95,563 (13.5) |
|  | 50-69, n (%) | 73,757 (10.4) |
|  | 70-79, n (%) | 35,279 (5.0) |
| **Cardiovascular outcome, n (%)** | | 25,280 (3.6) |
| **Death, n (%)** | | 38,984 (5.5) |
| **Composite outcome, n (%)** | | 57,456 (8.1) |
| **Follow up duration (years)** | | 10.7 ± 1.4 |
|  | | 11.0 [IQR 11.0-11.0] |

**
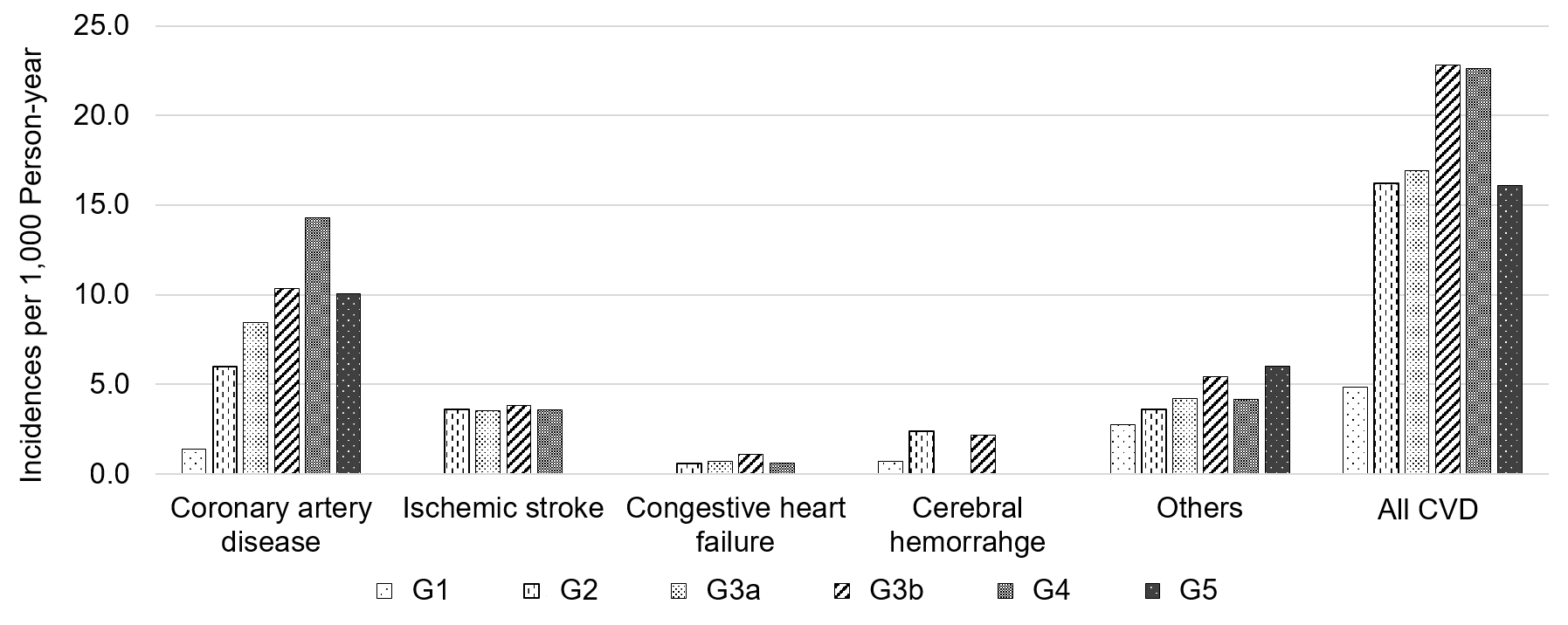
**

**Supplementary Figure S1. Incidence of cardiovascular disease in the KNOW-CKD cohort according to CKD stage**

CKD, chronic kidney disease; CVD, cardiovascular disease

With increasing CKD stage, an increasing incidence of coronary artery disease (events defined as myocardial infarction or unstable angina) and other CVD events was observed (*P* < 0.001 for both). For ischemic stroke, congestive heart failure, and hemorrhagic stroke events, however, statistical significance was not shown (*P* = 0.473, *P* = 0.524, and *P* = 0.425, respectively).


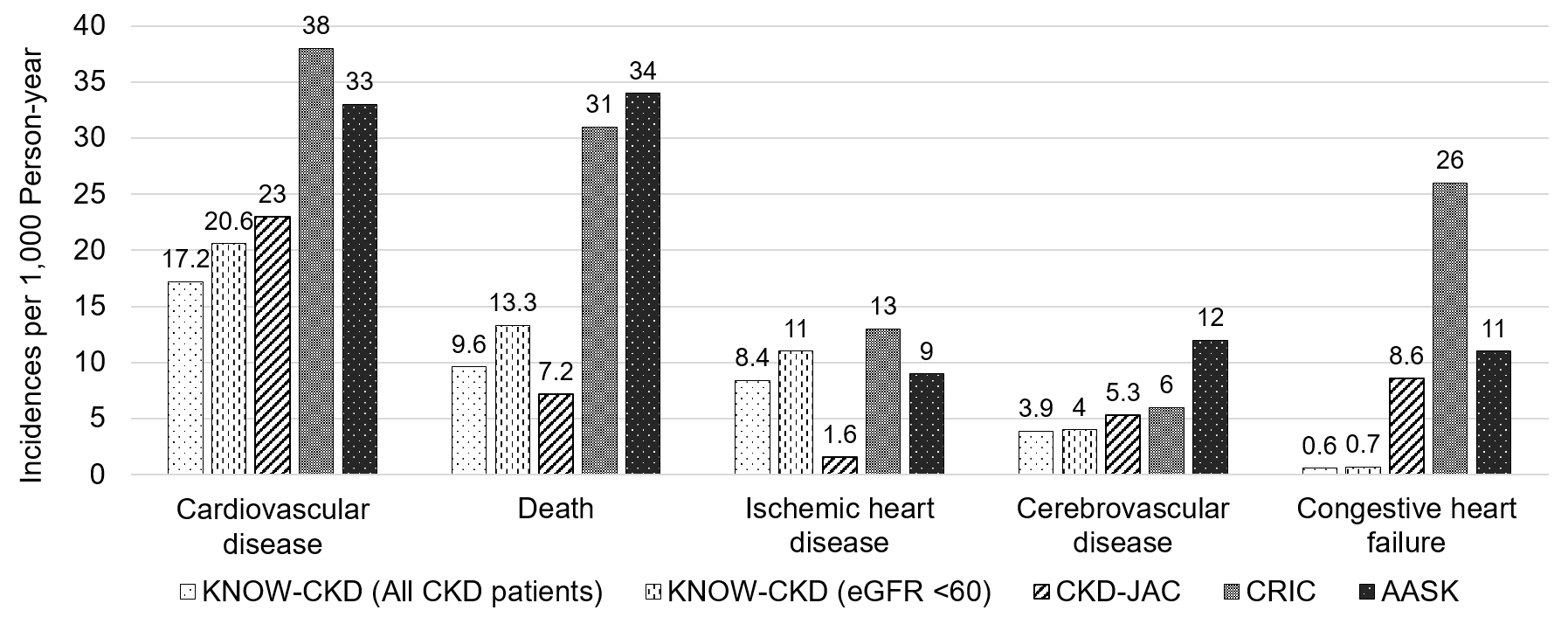


**Supplementary Figure S2. Incidence of outcomes compared to the other nations’ CKD cohorts**

AASK, African American Study of Kidney Disease and Hypertension; CKD, chronic kidney disease; CKD-JAC, Chronic Kidney Disease Japan Cohort; CRIC, Chronic Renal Insufficiency Cohort

The KNOW-CKD cohort population with CKD stage G3a-G5 showed a similar incidence of CVD and a higher incidence of death compared to the CKD-JAC cohort. Compared to the CRIC and AASK cohorts, the incidence of both CVD and death was lower in KNOW-CKD cohorts.

**Supplementary Table S1. Comparison of baseline clinical characteristics at enrollment with respect to the occurrence of composite outcomes**

|  | | **Total** | **Composite outcome (+)** | **Composite outcome (-)** | ***P*-value** |
| --- | --- | --- | --- | --- | --- |
| **Number** | | 2179 | 208 (9.5) | 1971 (90.5) |  |
| **Age (years)** | | 53.6±12.2 | 61.6 ± 78.7 | 52.8 ± 12.2 | <0.001 |
| **Female, n (%)** | | 843 (38.7) | 58 (27.9) | 785 (39.8) | 0.001 |
| **BMI, kg/m2** | | 24.6 ± 3.4 | 24.4 ± 2.9 | 24.6± 3.4 | 0.505 |
| **Systolic blood pressure (mmHg)** | | 128.5 ± 16.5 | 132.2 ± 19.5 | 128.1 ± 16.1 | 0.003 |
| **Diastolic blood pressure (mmHg)** | | 76.9 ± 11.1 | 75.9 ± 11.8 | 77.0 ± 11.1 | 0.182 |
| **Waist to hip ratio** | | 40.5 ± 6.1 | 40.1 ± 5.1 | 40.5 ± 6.2 | 0.280 |
| **Left ventricular mass index (g/m^2^)** | | 93.8 ± 25.1 | 103.4 ± 29.0 | 92.8 ± 24.5 | <0.001 |
| **Prevalence of cardiovascular disease, n (%)** | | 140 (6.4) | 44 (21.2) | 96 (4.9) | <0.001 |
| **Prevalence of diabetic mellitus, n (%)** | | 728 (33.4) | 107 (51.4) | 621 (31.5) | <0.001 |
| **Cause of chronic kidney disease** | |  |  |  | <0.001 |
|  | **Glomerulonephritis, n (%)** | 789 (36.2) | 37 (17.8) | 752 (38.2) |  |
|  | **Diabetic mellitus, n (%)** | 502 (23) | 87 (41.8) | 415 (21.1) |  |
|  | **Hypertension, n (%)** | 401 (18.4) | 38 (18.3) | 363 (18.4) |  |
|  | **Polycystic kidney disease, n (%)** | 357 (16.4) | 28 (13.5) | 329 (16.7) |  |
|  | **Others, n (%)** | 130 (6) | 18 (8.7) | 112 (5.7) |  |
| **eGFR (ml/min/1.73m^2^ )** | | 53.2 ± 30.7 | 41.0 ± 22.9 | 54.5 ± 31.2 | <0.001 |
| **Chronic kidney disease stage** | |  |  |  | <0.001 |
|  | **G1, n (%)** | 352 (16.2) | 9 (4.3) | 343 (17.4) |  |
|  | **G2, n (%)** | 415 (19) | 33 (15.9) | 382 (19.4) |  |
|  | **G3a, n (%)** | 359 (16.5) | 32 (15.4) | 327 (16.6) |  |
|  | **G3b, n (%)** | 459 (21.1) | 52 (25) | 407 (20.6) |  |
|  | **G4, n (%)** | 462 (21.2) | 64 (30.8) | 398 (18.3) |  |
|  | **G5, n (%)** | 132 (6.1) | 18 (8.7) | 114 (5.8) |  |
| **Hemoglobin (g/dL)** | | 12.8 ± 2.0 | 12.2 ± 2.0 | 12.9 ± 2.0 | <0.001 |
| **Uric acid (mg/dL)** | | 7.0 ± 1.9 | 7.3 ± 1.9 | 7.0 ± 1.9 | 0.015 |
| **Calcium (mg/dL)** | | 9.1 ± 0.5 | 9.0 ± 0.6 | 9.1 ± 0.5 | <0.001 |
| **Phosphorous (mg/dL)** | | 3.7 ± 0.7 | 3.8 ± 0.8 | 3.7 ± 0.7 | 0.053 |
| **Albumin (g/dL)** | | 4.2 ± 0.4 | 4.0 ± 0.5 | 4.2 ± 0.4 | <0.001 |
| **Total Cholesterol (mg/dL)** | | 174.0 ± 39.2 | 167.4 ± 39.7 | 174.7 ± 39.1 | 0.011 |
| **HDL-cholesterol (mg/dL)** | | 49.2 ± 15.3 | 46.4 ± 14.0 | 49.5 ± 15.4 | 0.005 |
| **LDL-cholesterol (mg/dL)** | | 96.9 ± 31.8 | 92.3 ± 30.0 | 97.4 ± 31.9 | 0.032 |
| **Triglyceride (mg/dL)** | | 157.3 ± 98.6 | 154.2 ± 87.4 | 157.7 ± 99.7 | 0.637 |
| **Fasting blood sugar (mg/dL)** | | 111.0 ± 39.8 | 118.5 ± 48.5 | 110.2 ± 38.7 | 0.018 |
| **HbA1C (%)**  **(Only in diabetic patients)** | | 7.2 ± 1.3 | 7.3 ± 1.1 | 7.2 ± 1.4 | 0.712 |
| **Intact parathyroid hormone (pg/mL)** | | 51.1 [32.3-84.0] | 61.6 [37.0-111.3] | 50.0 [31.7-81.1] | <0.001 |
| **Urine Protein/creatinine (g/g Cr)** | | 0.5 [0.1-1.5] | 0.9 [0.2-2.6] | 0.5 [0.1-1.4] | <0.001 |
| **High sensitive C-reactive protein (mg/dL)** | | 0.6 [0.2-1.7] | 0.9 [0.4-2.5] | 0.6 [0.2-1.6] | <0.001 |
| **Follow up duration (years)** | | 4.0 ± 1.7 | 3.84 ± 1.81 | 4.06 ± 1.72 | 0.091 |

Note: Values for categorical variables are shown as percentages; values for continuous variables, as mean ± standard deviation or median [interquartile range].

Abbreviations: BMI, body mass index; eGFR, estimated glomerular filtration rate; HbA1C, hemoglobin A1C; HDL, high-density lipoprotein; Hs-CRP, high sensitivity-C reactive protein; IQR, interquartile range; LDL, low-density lipoprotein

**Supplementary Table S2. Cause-specific hazard model for the multivariate regression analysis of outcomes according to CKD stage after adjustment for confounding factors (CKD stage 1 and 2 as a reference)**

| **Primary outcome: Cardiovascular disease** | | | | | | | | |
| --- | --- | --- | --- | --- | --- | --- | --- | --- |
|  | **Unadjusted** | | **Model 1** | | **Model 2** | | **Model 3** | |
| **CKD stages** | **HR (95%CI)** | ***P*-value** | **HR (95%CI)** | ***P*-value** | **HR (95%CI)** | ***P*-value** | **HR (95%CI)** | ***P*-value** |
| **Stage G1 and G2** | Reference |  |  |  |  |  |  |  |
| **Stage G3a** | 1.64 (0.97-2.77) | 0.065 | 0.97 (0.57-1.67) | 0.921 | 0.88 (0.51-1.51) | 0.650 | 0.86 (0.48-1.52) | 0.595 |
| **Stage G3b** | 2.30 (1.46-3.62) | <0.001 | 1.31 (0.81-2.10) | 0.266 | 1.08 (0.66-1.75) | 0.771 | 1.11 (0.66-1.86) | 0.695 |
| **Stage G4** | 2.72 (1.70-4.34) | <0.001 | 1.44 (0.88-2.36) | 0.147 | 1.15 (0.70-1.90) | 0.584 | 1.14 (0.66-1.98) | 0.628 |
| **Stage G5** | 3.1 (1.42-6.75) | 0.004 | 1.86 (0.84-4.13) | 0.126 | 1.72 (0.78-3.80) | 0.181 | 1.64 (0.69-3.87) | 0.261 |
| **Secondary outcome: Composite outcome of cardiovascular disease and all-cause death** | | | | | | | | |
|  | **Unadjusted** | | **Model 1** | | **Model 2** | | **Model 3** | |
| **CKD stages** | **HR (95%CI)** | ***P*-value** | **HR (95%CI)** | ***P* value** | **HR (95%CI)** | ***P*-value** | **HR (95%CI)** | ***P*-value** |
| **Stage G1 and G2** | Reference |  |  |  |  |  |  |  |
| **Stage G3a** | 1.80 (1.13-2.85) | 0.013 | 1.07 (0.66-1.71) | 0.791 | 0.97 (0.60-1.56) | 0.903 | 0.96 (0.58-1.58) | 0.858 |
| **Stage G3b** | 2.33 (1.55-3.50) | <0.001 | 1.34 (0.87-2.04) | 0.181 | 1.13 (0.73-1.74) | 0.583 | 1.21 (0.77-1.92) | 0.408 |
| **Stage G4** | 3.92 (2.65-5.82) | <0.001 | 2.05 (1.35-3.11) | <0.001 | 1.69 (1.11-2.59) | 0.015 | 1.78 (1.12-2.81) | 0.014 |
| **Stage G5** | 5.91 (3.38-10.33) | <0.001 | 3.35 (1.88-5.98) | <0.001 | 3.08 (1.73-5.48) | <0.001 | 3.15 (1.71-5.83) | <0.001 |

Model 1: adjusted for age and sex

Model 2: adjusted for the variables in model 1 as well as the prevalence of diabetes, body mass index, systolic blood pressure, and prevalence of cardiovascular disease

Model 3: adjusted for the variables in model 2 as well as left ventricular mass index, serum hemoglobin, serum uric acid, serum albumin, LDL cholesterol, fasting blood sugar, urine protein-to-creatinine ratio, high-sensitivity C-reactive protein, and intact parathyroid hormone

Abbreviations: CI, confidence interval; CKD, chronic kidney disease; HR, hazard ratio

**Supplementary Table S3. Age- and sex-adjusted hazard ratios according to CKD stage in subgroup analysis using a cause-specific hazard model (CKD stages G1-G2 as a reference)**

|  | | **Cardiovascular disease** | | | | **Composite outcome** | | | |
| --- | --- | --- | --- | --- | --- | --- | --- | --- | --- |
|  | | **CKD stage G3a-3b** | | **CKD stage G4-5** | | **CKD stage G3a-3b** | | **CKD stage G4-5** | |
| **CKD stage G1 and G2 as reference** | | **HR (95% CI)** | ***P*-value** | **HR (95% CI)** | ***P*-value** | **HR (95% CI)** | ***P*-value** | **HR (95% CI)** | ***P*-value** |
| **Age** | **<55 years** | 1.44 (0.62-3.34) | 0.403 | 1.94 (0.70-5.46) | 0.205 | 1.46 (0.73-2.92) | 0.289 | 2.24 (0.96-5.21) | 0.061 |
|  | **≥ 55 years** | 1.08 (0.66-1.78) | 0.760 | 1.42 (1.31 1.72) | 0.198 | 1.17 (2.94 3.86) | 0.517 | 2.26 (1.41-3.62) | <0.001 |
| **Sex** | **Male** | 1.34 (0.78-2.27) | 0.287 | 1.65 (0.50 2.03) | 0.095 | 1.40 (0.48 1.71) | 0.170 | 2.48 (1.51-4.08) | <0.001 |
|  | **Female** | 0.87 (0.39-1.91) | 0.722 | 1.34 (1.14 2.24) | 0.494 | 0.91 (1.96 3.86) | 0.796 | 1.95 (0.97-3.92) | 0.06 |
| **Diabetes Mellitus** | **With** | 1.03 (0.53- 2.01) | 0.932 | 1.17 (0.84 1.67) | 0.669 | 1.24 (2.23 4.45) | 0.510 | 2.04 (1.06-3.93) | 0.033 |
|  | **Without** | 1.03 (0.57-1.84) | 0.931 | 1.40 (1.10 1.89) | 0.324 | 1.01 (2.99 5.13) | 0.968 | 1.94 (1.14-3.31) | 0.015 |
| **UPCR** | **< 1g/g Cr** | 1.44 (0.84-2.47) | 0.191 | 1.32 (0.68-2.56) | 0.409 | 1.24 (0.76-2.03) | 0.390 | 1.75 (1.01-3.02) | 0.046 |
|  | **≥ 1g/g Cr** | 0.602 (0.28-1.28) | 0.189 | 1.00 (0.48-2.10) | 0.998 | 0.85 (0.45-1.62) | 0.614 | 1.76 (0.94-3.29) | 0.079 |
| **CKD causes** | **Glomerulonephritis** | 1.04 (0.45-2.40) | 0.922 | 1.16 (0.41-3.23) | 0.781 | 1.18 (0.55-2.52) | 0.672 | 1.56 (0.64-3.80) | 0.325 |
|  | **Diabetic nephropathy** | 0.77 (0.35-1.72) | 0.527 | 1.01 (0.44-2.31) | 0.989 | 0.94 (0.43-2.06) | 0.876 | 1.75 (0.80-3.83) | 0.160 |
|  | **Hypertensive nephropathy** | 0.97 (0.27-3.47) | 0.958 | 1.69 (0.46-6.22) | 0.429 | 0.98 (0.33-2.95) | 0.972 | 1.81 (0.59-5.52) | 0.300 |
|  | **Polycystic kidney disease** | UC | UC | UC | UC | 2.48 (1.00-6.12) | 0.049 | 4.74 (1.55-14.52) | 0.007 |
|  | **Others** | 0.83 (0.25-2.78) | 0.764 | 1.44 (0.37-5.62) | 0.596 | 0.72 (0.23-2.28) | 0.577 | 2.04 (0.62-6.66) | 0.239 |

Abbreviations: CI, confidence interval; CKD, chronic kidney disease; HR, hazard ratio; UC, unable to calculate; UPCR, urine protein-to-creatinine ratio
